# Motile Sperm Output by Male Cheetahs (*Acinonyx jubatus*) Managed *Ex Situ* Is Influenced by Public Exposure and Number of Care-Givers

**DOI:** 10.1371/journal.pone.0135847

**Published:** 2015-09-02

**Authors:** Diana C. Koester, Elizabeth W. Freeman, Janine L. Brown, David E. Wildt, Kimberly A. Terrell, Ashley D. Franklin, Adrienne E. Crosier

**Affiliations:** 1 Center for Species Survival, Smithsonian Conservation Biology Institute, Front Royal, Virginia, United States of America; 2 Department of Environmental Science and Policy, George Mason University, Fairfax, Virginia, United States of America; 3 New Century College, George Mason University, Fairfax, Virginia, United States of America; 4 Department of Ecology and Evolutionary Biology, Tulane University, New Orleans, Louisiana, United States of America; 5 Department of Animal and Avian Sciences, University of Maryland, College Park, Maryland, United States of America; Federal University of Parana (UFPR)) – Campus Palotina, BRAZIL

## Abstract

The collective cheetah (*Acinonyx jubatus*) population in zoological institutions has never been self-sustaining because of challenges in natural reproduction. A retrospective analysis of North American zoo-breeding records has revealed that >90% of litters produced since 2003 occurred in facilities ‘off-display’ from the public. We examined seminal, endocrine, and behavioral traits of 29 adult male cheetahs that were: 1) managed in public exhibit or off-display facilities; 2) maintained by different numbers of cheetah-specific care-givers; and 3) living adjacent to varying numbers of adult conspecifics. Cheetahs housed off-display produced more total motile sperm/ejaculate (*P* = 0.04) than on-exhibit males. This finding was mirrored in our laboratory’s historical records where two-fold more total motile sperm (*P* < 0.01) were measured in ejaculates from individuals with no public exposure (n = 43) compared to on-exhibit (n = 116) counterparts. Males at institutions with ≤3 care-givers also produced more total motile sperm/ejaculate (*P* < 0.03) and spent more time behaviorally active (*P* < 0.01) than at facilities using >3 care-givers. Exposure to high numbers of conspecifics within the same institution did not impact (*P* > 0.05) seminal traits, and presence of the public, care-giver number, or animals/facility had no influence (*P* > 0.05) on androgen or glucocorticoid excretion or other behavioral metrics. Findings indicate that male cheetahs are sensitive to general public exposure and too many care-givers, resulting in compromised motile sperm output/ejaculate with mechanism of action unrelated to altered androgen or glucocorticoid excretion.

## Introduction

The wild cheetah (*Acinonyx jubatus*) population is in decline with only ~10,000 individuals remaining in nature today [1]. Free-living individuals of this species are challenging to study due to low population density *in situ* and elusive behaviors. Thus, much of what is known about the specifics of cheetah physiology, endocrinology, immunology, and sensitivity to disease has been learned from animals managed in *ex situ* ‘insurance’ populations, mostly in breeding centers and zoos. Besides being useful for generating new information on species biology, these collections inspire the public through zoo exhibits and education programs. But the *ex situ* population has never been self-sustaining, that is, reproductively, demographically, and genetically viable [2, 3]. Even with involvement of major zoological institutions accredited by the Association of Zoos & Aquariums (AZA), only ~20% of cheetahs in the North American population have ever reproduced [4].

There has been 30 years of debate on the causes for the cheetah’s low reproductive performance. The species is well recognized for its lack of genetic variation [5, 6] and the production of ejaculates containing low sperm concentrations and high proportions of sperm malformations [7–11]. Although these structurally-impaired cells do not participate in fertilization [10, 12, 13], males with these seminal traits sire litters, even after a single copulatory episode [2], and there is no evidence for low fertility in cheetahs free-living in Africa despite displaying poor sperm morphology and ejaculate quality similar to captive counterparts [11, 14]. Therefore, animal management practices have been explored as potential causes of the suboptimal reproductive performance. Physiological stress has been one target with Terio et al., 2004 [15] suggesting that cheetahs managed in captivity excrete higher glucocorticoid concentrations than wild counterparts. A related study determined that moving cheetahs from off-public breeding centers into urban zoos increased excreted glucocorticoid levels [16]. Another report demonstrated that number of adult female cheetahs in a given place could impact ovarian activity. Specifically, Wielebnowski and colleagues [17] determined that pairs of incompatible cheetahs living in the same enclosure could mutually suppress ovarian cyclicity, an inhibition reversible when females were separated, even by a simple fenceline and continued visual and olfactory proximity. Collectively, these findings appear consistent with the natural history of wild cheetahs where females largely remain solitary [18]. These observations have motivated some zoological institutions to develop spacious, off-site breeding centers that are mostly off limits to the visiting public. This tactic also appears significant in that, from 2003 through 2013, >90% of cheetah litters have been produced in these off-exhibit centers compared to urban zoos [4, 19].

Therefore, there is some evidence for greater reproductive success for managed cheetahs in the presence of more space, fewer conspecifics in the facility, and perhaps less overall exposure to people. Although hyper-adrenal activity may be involved, increased acute or chronic glucocorticoid patterns have not been linked to any specific reproductive trait in this species. Because most studies to date have been skewed towards females, the male becomes an attractive investigative target because of its thoroughly studied semen phenotype and well-described procedure to measure sperm quality [7, 9, 11, 20]. There also are established species ethograms for behavioral metrics [17, 21, 22] and the ability to non-invasively assess androgen and glucocorticoid metabolite patterns in feces [15–17, 23, 24] as measures of testicular and adrenal (stress) status, respectively [25, 26].

Using these tools, our aim was to determine the influence of both people and conspecifics on cheetah seminal, endocrine, and behavioral values under *ex situ* conditions. We hypothesized that cheetahs managed (1) ‘off-exhibit’ compared to ‘on-exhibit’ (with nil vs. high public exposure, respectively) or (2) under the care of a limited number of specialist care-givers would experience improved sperm quality, higher androgen and lower glucocorticoid metabolite patterns, and more normative, ‘active’ behaviors. Our second hypothesis considered the solitary nature of some members of this species in the wild and postulated that the same enhancements would be experienced in facilities holding fewer conspecifics. As a few other felid species demonstrate variations in ejaculate quality through the year [27, 28], we examined seminal traits and androgen metabolite patterns over time to ensure that seasonality was not confounding results. Our analysis was bolstered with a parallel examination of our historical database that included more than 80 additional cheetahs housed in on- or off-exhibit conditions within the USA and evaluated using the same seminal collection methods as in the present study. The by-product was the first-ever hormonal evaluation of the presence/absence of male cheetah seasonality.

## Materials and Methods

### Animals and management conditions

Adult, male cheetahs (n = 29) were housed at seven AZA-accredited institutions in the USA (Table 1). Animals ranged from 2 to 12 yr of age (mean ± standard error of the mean [SEM], 6.9 ± 0.5 yr), a range considered as reproductively mature [7, 11, 19]. All cheetahs had been born in captivity and managed according to husbandry guidelines established by AZA’s Cheetah Species Survival Plan (SSP) Program [4, 21]. Cheetahs lived in enclosures from 0.03 to 0.60 ha in size. Diet included feeding a commercial meat product (beef or horse-based) at least 5 d/wk with occasional supplements of whole rabbit, bones (beef, horse, bison, venison), or organ meat (beef, horse, venison); water was available *ad libitum*.

**Table 1:** Demographic and housing information for male cheetahs (n = 29).

Facility	State	Number of males		
		Single	Grouped	Total
Dickerson Park Zoo	MO	1	0	1
Philadelphia Zoo	PA	0	3	3
San Diego Safari Park	CA	1	5 (1 pair, 1 trio)	6
Smithsonian Conservation Biology Institute	VA	1	6 (2 trios)	7
White Oak Conservation Center	FL	0	4 (2 pairs)	4
Wildlife Safari	OR	2	2	4
The Wilds	OH	1	3	4

Study cheetahs were categorized in three ways, the first based on living in ‘on-exhibit’ or ‘off-exhibit’ facilities. Animals in visual contact with >50 different person visitors to the institution per month for ≥5 consecutive months were classified as living on-exhibit. Secondly, the study group was characterized on the basis of number of care-givers (keepers) for a given institution’s collection. Keeper number was defined as number of persons having direct interaction with these specific animals during daily work duties that included feeding, cleaning enclosures, shifting animals within the facility, and providing behavioral training and enrichment. Not all care-givers interacted with the collection on a daily basis. Rather, this condition reflected the total number of keepers participating in the overall care of that particular collection. Finally, study group also was examined on the basis of number of conspecific animals within a given facility, specifically number of other male or female cheetahs ≥2 yr of age in visual and/or olfactory range to the test male(s).

### Ethics statement

This study was carried out in strict accordance with recommendations in the Guide for the Care and Use of Laboratory Animals of the National Institutes of Health. The Smithsonian National Zoological Park’s Animal Care and Use Committee approved this study (permit numbers: 09–41, 13–44).

### Ejaculate collection and evaluation

Semen was collected one to two times/male beginning at least 2 mo after fecal sampling commenced for endocrine monitoring (see below). The process was by electroejaculation [7, 8, 11, 20, 29] that involved fasting the cheetah for approximately 24 h before inducing surgical anesthesia using an i.m. injection of a combination of the following four drugs, (as deemed most appropriate by the attending veterinarian): medetomidine hydrochloride, midazolam, and/or butorphanol (Domitor, Pfizer Inc., La Jolla, CA; 22.0–25.0 μg/kg body weight, 0.2 mg/kg, and 0.3 mg/kg, respectively), and/or ketamine HCl (Ketaset; Fort Dodge Laboratories, Fort Dodge, IA; 2.0–3.5 mg/kg). Propofol (PropoFlo, 0.5–4.0 mg/kg, i.v.) was administered, as necessary, to maintain an appropriate plane of anesthesia during electroejaculation. Once each animal was fully anesthetized, testicular dimensions were measured using calipers and results converted to volume [30]. Each ejaculate collection and subsequent evaluation were performed according to previously described, rigorous protocols and metrics [7, 11, 29, 30] and by a single individual to avoid bias. Briefly, this involved using a standardized set of three stimulation series over an approximate 30 min interval to collect cheetah semen into sterile vials. At the end of each series, a 3 μl seminal aliquot was examined microscopically for sperm percent motility and forward progressive status (0–5 scale, 5 being rapid, straightforward sperm progress over 5 microscopic fields at 200x). At the end of all collection series, semen was combined into a single sample, mixed, and volume measured. A 15 μl aliquot was fixed in 100 μl of a 0.3% glutaraldehyde in phosphate buffered saline solution (pH, 7.4) for subsequent evaluations of sperm morphology (1000x). For this process, 100 spermatozoa/ejaculate were individually examined and classified as normal or having one of the structural malformations typical for this species [7, 9, 11, 20, 29, 31]. Sperm concentration in the ejaculate was determined using a standard hemocytometer technique [29].

### Fecal collection and preparation

Freshly voided feces were collected 3 to 4 d/wk for 7 to 13 mo/male. Approximately 50 g of each sample were placed in a labeled, plastic bag and stored at -20°C until analyzed at the Smithsonian Conservation Biology Institute (SCBI) for hormonal metabolites. When males were managed together in the same enclosure, a different non-digestible and harmless marker (e.g., non-toxic glitter, birdseed, lentils, corn) was added to each food pan so that a fecal sample could be traced back to the appropriate donor. Fecal samples (n = 4,653) were individually lyophilized (VirTis, 35L Ultra Super XL-70, Gardiner, NY), then pulverized using a mallet, and stored at -20°C until processed further.

Steroid hormonal metabolites were extracted from each sample using previously described methods [32]. Efficiency of steroid extraction was evaluated by adding radiolabeled hormone (^3^H-testosterone or ^3^H-cortisol; 4,000–8,000 dpm) to each sample prior to boiling extraction. The overall mean (± SEM) extraction efficiency for all samples was 78.2% ± 0.11%. Fecal extracts were diluted 1:20 or 1:200 in BSA-free phosphate buffer (2.2 M NaH_2_PO_4_, 3.5 M Na_2_HPO_4_, 0.3 M NaCl, H_2_O; pH, 7.0) for androgen and glucocorticoid enzyme immunoassay (EIA) analysis, respectively. Fecal hormone data were expressed as μg/g dry feces.

### Fecal androgen metabolite analysis

The testosterone EIA relied on a polyclonal anti-testosterone antibody (R156/7; C. Munro, University of California, Davis, CA) [33] that cross-reacted with testosterone (100%), 5α-dihydrotestosterone (57.4%), <1% with androstenedione, androsterone, androsteneolone, cholesterol, and estradiol-17β, and <0.02% with progesterone, pregnenolone, and hydrocortisone. Antibody (0.05 ml) was added to 96-well microtiter plates (Nunc-Immuno, Maxisorp; Fisher Scientific) and equilibrated for 12 to 48 h (4°C). Unbound antibody was removed with wash solution (1.5 M NaCl, 0.06 mM, 5.0 mL Tween 20 [Sigma-Aldrich, P1379]), and diluted samples (in duplicate) and standards (in triplicate) (0.05 ml; 46–12,000 pg/ml; 17β-hydroxy-4-androstein-3-one; Steraloids) were added to the plate. A peroxidase enzyme-conjugated testosterone (1:15,000; 0.05 ml; C. Munro) then was added to each well containing sample or standard and the plate incubated for 2 h (23°C) before unbound components were removed with wash solution. A chromagen solution (0.1 ml; 2,2'-azino-di-[3-ethyl-benzthiazoline-6-sulphonic acid], ABTS) then was added to each well and incubated ~20 min before optical densities were determined using a microplate reader (Dynex MRX, reading filter at 405 nm, reference filter at 540nm). Samples considered too dilute (binding >80% of maximum) were run at a higher concentration (1:100), and samples that were too concentrated (binding <20%) were run at a lower concentration (1:2,000).

Sensitivity of the testosterone EIA at maximum binding was 2.3 pg/well. The inter-assay coefficient of variation for two internal controls was 10.6% (mean binding, 24.1%) and 5.0% (mean binding, 66.2%), and the intra-assay coefficient of variation was <10% (n = 326 assays). Serially diluted, pooled fecal extracts expressed displacement curves parallel to those of the testosterone standard curve. Recovery of added testosterone to fecal extract demonstrated significant recovery (y = 1.08x − 3.80, *r* = 0.99; *P* < 0.05). A detailed description and related results for the testosterone EIA validation is provided in S1 Text and S1 Fig.

### Fecal glucocorticoid metabolite analysis

Glucocorticoid metabolite concentrations in diluted extracts were determined using a cortisol EIA validated for use in the cheetah [23]. The polyclonal antibody used (R4866; C. Munro) had been raised in rabbits against cortisol-3-carboxymethyloxime linked to bovine serum albumin. The antibody cross-reacted with cortisol (100%), prednisolone (9.9%), prednisone (6.3%), cortisone (5%), and <1% with corticosterone, desoxycorticosterone, 21-desoxycortisone, testosterone, androstenedione, androsterone, and 11-desoxycortisol [23]. Microtiter plates were run as described above for androgen analysis; diluted samples in duplicate, standards in triplicate (0.05 ml; 78–20,000 pg/ml; Sigma Diagnostics), and a peroxidase enzyme-conjugated cortisol tracer (1:20,000; 0.05 ml; C. Munro) were added to the plate. The latter was incubated for 1 h (23°C) before unbound components were removed, ABTS added, and optical densities read. Samples considered too dilute (binding >80% of maximum binding) were run at a higher concentration (1:10), and samples too concentrated (binding <20%) were run at a lower concentration (1:200).

Sensitivity of the EIA was 3.9 pg/well at maximum binding. The inter-assay coefficient of variation for two internal controls was 8.4% (mean binding, 27.9%) and 4.8% (mean, 68.1%) whereas the intra-assay coefficient of variation was <10% (n = 364 assays). Serially diluted, pooled, fecal extracts expressed displacement curves parallel to those of the cortisol standard curve. Recovery of added cortisol standard to fecal extract demonstrated significant recovery (y = 0.90x − 18.02, *r* = 0.99; *P* < 0.05). A description and the results associated with the cortisol EIA validation are provided in S2 Text, S2 Fig, and S1 Table.

### Behavioral observations

Behavioral evaluations were conducted on 8 consecutive days during the last 6 mo of the fecal collection period. Males living in pairs or trios were observed for 1 h and singletons for 30 min at some point in time from 0600 to 1000 h in the ‘home’ enclosure. This specific sampling was used to establish baseline behavioral frequencies. Instantaneous sampling (2 min for coalition males and 1 min for singletons) was used to estimate time spent in any behavioral state, and continuous scan sampling was used to measure behavioral event frequencies [34]. Behavioral state data were converted to the proportion of time each male spent/h in each state. Behavioral event frequencies were reported as average frequency/h for each male for each behavior [34]. These proportions of time/h and frequency/h values for each male were used to examine the relationship between behavior and our three management factors of interest. Observations were recorded using methods and definitions of observable behaviors (Table 2) described in the Cheetah Husbandry Manual [21] and consistent with ethograms and methods used previously by our laboratory [17, 22]. To ensure consistency, a single researcher made all behavioral observations.

**Table 2:** Definitions of observable state and event behaviors for male cheetahs.

Behavioral states	
Resting	Side of body on ground, legs to side, eyes open or closed
Standing	As implied
Crouching	Legs under body like standing, but legs bent, body close to ground
Sitting	As implied
Walking	Forward locomotion at relaxed speed, that includes trot
Pacing	Walking back and forth across same area repeatedly
Running	Rapid forward locomotion with full extension of limbs and tail
Out of Sight	Animal unable to be seen by observer to assess state
Behavioral events	
Rub	Rubs face, head, or neck on object or conspecific
Roll	Rolls from one side to another, back on ground, paws in the air
Sniff	Olfactory examination of ground, object, or conspecific
Groom	Lick, chew, or otherwise clean self or conspecific
Stutter	Repetitive short throat calls
Meow	Soft call, similar to that of domestic cat
Chirp	High pitched, short call, corners of mouth drawn back
Growl	Low, drawn out ‘snarling’ sound, mouth open showing teeth
Tread	Scraping ground with rear legs without forward locomotion
Urinate	Excretion of urine from a sitting or squat position
Urine Spray	Sprays urine directly behind from standing position with tail raised
Defecate	As implied

### Statistical analysis

To test our hypotheses, the influence of the factors on- versus off-exhibit, number of keepers, and number of cheetah conspecifics on sperm, hormonal, and behavioral metrics were analyzed using a mixed model ANOVA. Besides exhibit status, we also considered location (institution) and animal grouping (singleton versus groups of 2 or 3 males nested within location) as fixed factors, with age and number of conspecifics present as covariates. Non-orthogonal linear contrasts were used to determine impact of exhibit status, number of keepers (institutions with ≤3 or >3 keepers on staff), and number of conspecifics (≤11 conspecifics or >11 conspecifics of either sex held in nearby enclosures). These specific numerical cutoffs for number of keepers and conspecifics were selected because sample size and distribution of males in this study did not provide sufficient statistical power for regression analysis of these two factors on male reproductive metrics. Therefore, distinct numerical category cutoffs were selected (i.e., ≤3 or >3 keepers; ≤11 or >11 conspecifics) to cluster males evenly into two groups for each factor for statistical analysis.

When examining the distribution of males between factor categories, an approximately equal number of males in each the ≤3 and >3 keeper categories were present in both exhibit status and number of conspecific categories, allowing keeper number to be analyzed within the model as independent from the other two factors. The influence of exhibit status and number of conspecifics on reproductive metrics, however, was confounded in the model because most off-exhibit facilities managed more cheetahs than on-exhibit institutions. To separate these effects, post-hoc analyses were performed when linear contrasts for exhibit status and/or ≤11 or >11 conspecifics were found to be significant (*P* < 0.05) [35]. For post-hoc evaluation of significant variables only, and to control for the effect of grouping, we analyzed only group-housed males (n = 23). This approach balanced the datasets so that numbers of males in each conspecific category were approximately equal for each exhibit status and vice versa. Reduction of the dataset resulted in heteroscedasticity, and, therefore, nonparametric Mann-Whitney U tests were used for post-hoc comparisons.

Prior to analysis with the mixed model to test our hypotheses, statistical outliers defined by Hoaglin and Iglewicz [36] were identified and replaced with the nearest neighboring value [37] for all semen quality (<3% of data) and hormone (<7% of data) values. There were no outliers in behavioral data. Normality and homoscedasticity were verified using an Anderson-Darling and a Levene’s test, respectively [35]. Linear associations among sperm, hormonal, and behavioral metrics were evaluated using correlation analyses (Pearson’s) [35]. Effects were considered significant at *P* < 0.05, and data were reported as mean ± SEM. All statistical procedures were conducted using SAS software (version 9.2, Cary, NC).

After normality and homoscedasticity checks, all semen quality metrics were analyzed in the mixed model for relationships to our three factors of interest. For all males, three semen quality metrics: total sperm, total motile sperm, and sperm concentration were not normal, but each passed normality testing after a log transformation. Therefore, log transformed data were used for evaluating these metrics. We also took advantage of an already existing 32 year long dataset (1981–2013) from our laboratory that included another 140 ejaculates from 85 cheetahs housed in the USA and where collections/evaluations were made in the same fashion. As our assessments in both the contemporary study and the historic database were conducted throughout the year, we also took the opportunity to assess information on the basis of seasonality. For this assessment, we used typical solstice and equinox dates for the temperature zone (i.e., winter = Dec 21–Mar 19; spring = Mar 20–Jun 20; summer = Jun 21–Sep 21; fall = Sep 22–Dec 20). A one-way ANOVA with Tukey-Kramer multiple mean comparison tests was used to examine sperm metrics from both the present and historic databases across seasons. Because the living status of cheetahs contributing to the historical database was known (116 ejaculates from 72 cheetahs on-exhibit versus 43 ejaculates from 30 cheetahs off-exhibit), sperm metrics from these two populations also were compared using a Student’s *t*-test [35].

To analyze hormonal metrics in the mixed model, two measures were calculated for fecal androgen and glucocorticoid data across cheetahs: 1) an overall mean of all samples for the entire collection period per male; and 2) a baseline that was calculated via a previously described iterative process [32] that determined the mean of all values excluding those greater than the overall mean plus 1.5 standard deviations (SD). A glucocorticoid peak frequency also was calculated for each male using a previous approach [23] that involved dividing the number of samples greater than three times baseline by total number of samples collected for that individual. To determine if season of the year influenced either androgen or glucocorticoid levels or patterns, a repeated measures ANOVA was used followed by a Tukey-Kramer multiple mean comparison test with an unstructured covariance matrix [35].

Principal component analysis (PCA) was used to identify correlated groups of state and event behaviors to reduce number of behavioral variables to be analyzed [34] with respect to our three main factors of interest. First, raw behavioral events ‘meow’ and ‘chirp’ were combined into a single ‘meowchirp’ category as these vocalizations often occurred together and were not always distinguishable by the observer. Resulting principal components with eigenvalues greater than 1 were retained, labeled according to the variables that showed the highest loadings, and these component scores then were analyzed in the statistical model as behavioral metrics [38]. To aid visual interpretation of findings, raw behavioral observation results were combined according to PCA clusters and displayed in figures with the statistical output from component scores.

## Results

### Ejaculate evaluation

**Fig 1:**
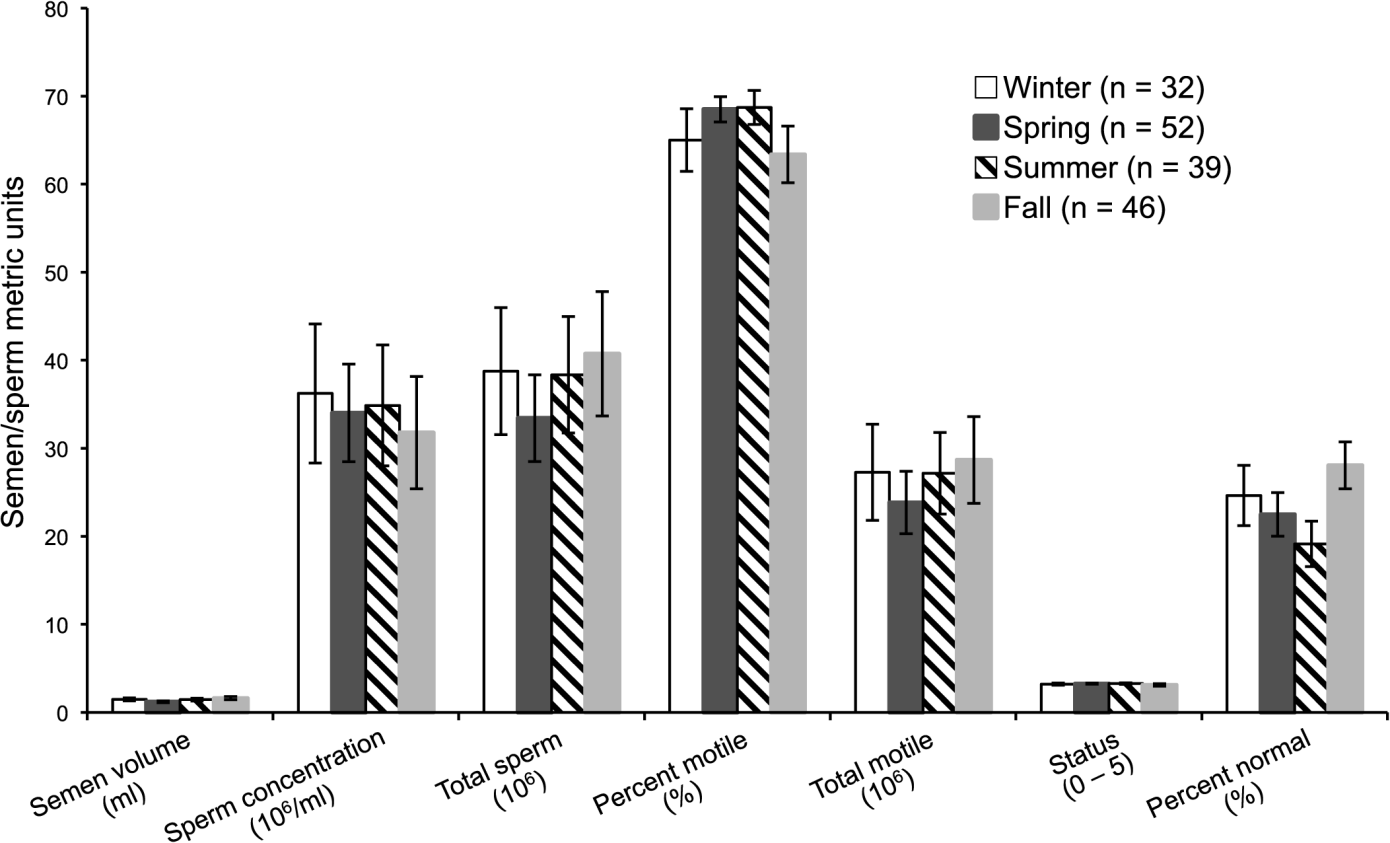
Mean (± SEM) seasonal ejaculate traits from cheetahs where semen was collected in different seasons at institutions throughout the USA (169 total ejaculates; number/season indicated in parentheses). There were no differences (*P* > 0.05) in any trait across seasons.

**Fig 2:**
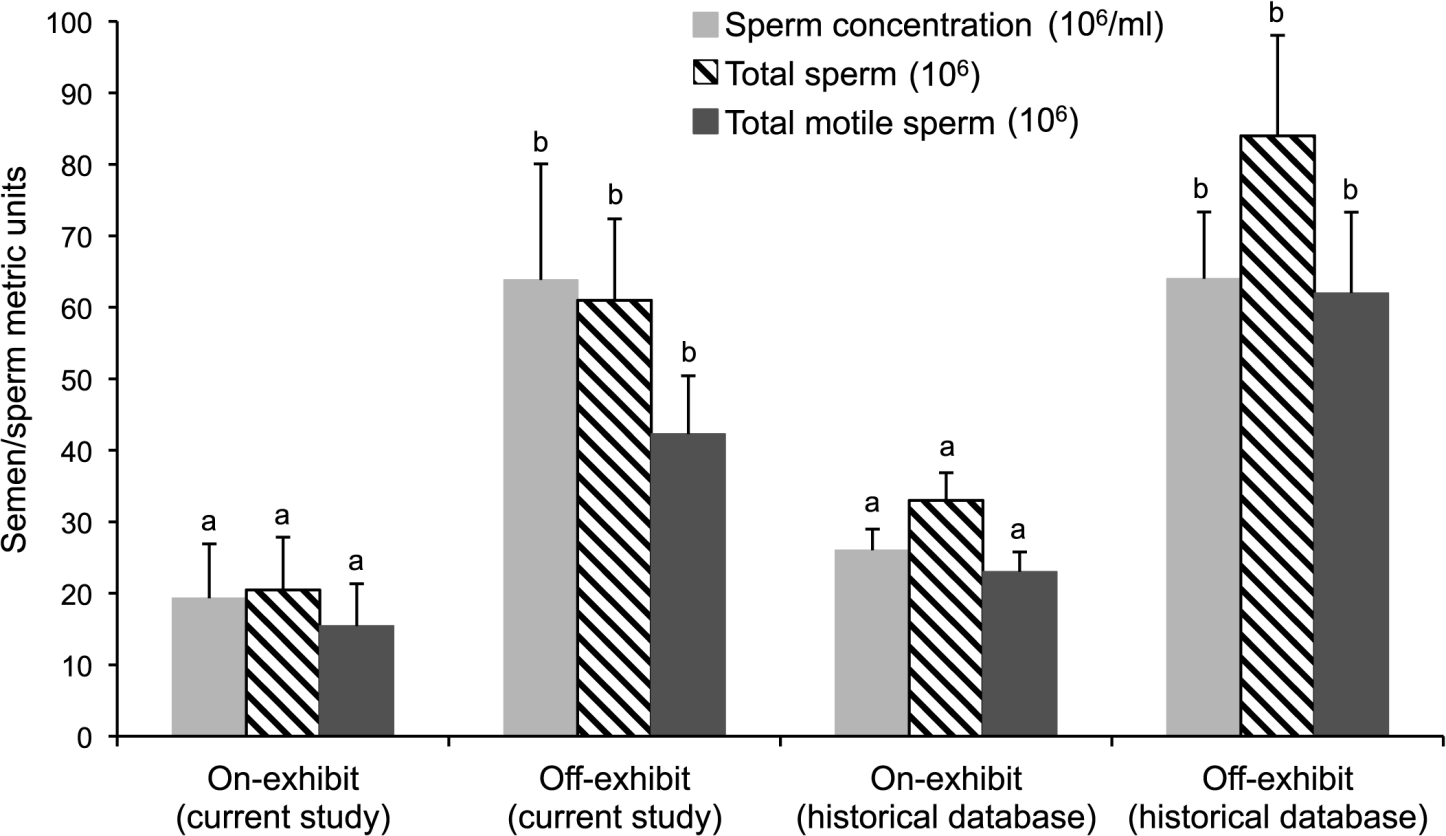
Mean (± SEM) ejaculate traits for male cheetahs (n = 23) managed in *ex situ* collections in the current study or from those in our institution’s historical database (n = 159 ejaculates) that were housed on- or off-exhibit. Superscripts represent differences (*P* < 0.05) in ejaculate metrics between the two conditions (on- or off-display) within the current or historical dataset.

There was no influence (*P* > 0.05) of season on seminal metrics for males in this study and those in our historical database (Fig 1). Post-hoc analysis revealed that males maintained in off-exhibit conditions produced ejaculates with higher sperm concentration (63.8 ± 16.2 x 10^6^/ml; *U* = 213.0; *P* = 0.03), more total spermatozoa (61.0 ± 11.4 x 10^6^ cells; *U* = 215.0; *P* = 0.02), and total motile spermatozoa (42.3 ± 8.2 x 10^6^; *U* = 211.0; *P* = 0.04) than on-exhibit counterparts (19.3 ± 7.62 x 10^6^/ml, 20.5 ± 7.4 x 10^6^, 15.4 ± 5.9 x 10^6^, respectively). The magnitude of these differences is best illustrated in Fig 2 that also demonstrates that these same sperm metrics differed (*P* < 0.01) between on- versus off-display cheetahs in the historical database. Other traits evaluated, including testicular volume, seminal volume, the two cell motility traits, and the proportions of total malformed spermatozoa (S2 Table) and specific structural deformities (S3 Table) were not different (*P* > 0.06) between the on- versus off-exhibit treatment conditions.

When males lived at institutions where care-giving was provided by ≤3 keepers, ejaculates contained more total (26.9 ± 1.3 x 10^6^; t_16_ = 2.69; *P* = 0.02) and total motile (17.2 ± 1.3 x 10^6^; t_16_ = 2.45; *P* = 0.03) spermatozoa than in cases where facilities relied on >3 keepers (20.0 ± 1.4 x 10^6^, 13.7 ± 1.4 x 10^6^, respectively). This treatment factor had no effect (*P* > 0.05) on testicular or other seminal metrics, including sperm concentration per ejaculate (t_16_ = 2.05; *P* = 0.06). Additionally, there were no differences (*P* > 0.05) in testicular volume or any ejaculate trait, including sperm concentration (*U* = 125.5; *P* = 0.71) and total motile sperm (*U* = 144.0; *P* = 1.00) between males housed at institutions with ≤11 versus >11 conspecifics present (S3 Fig). Our statistical model found no influence (*P* > 0.05) of the covariate individual male age on any analyzed trait.

### Endocrine evaluations

**Fig 3:**
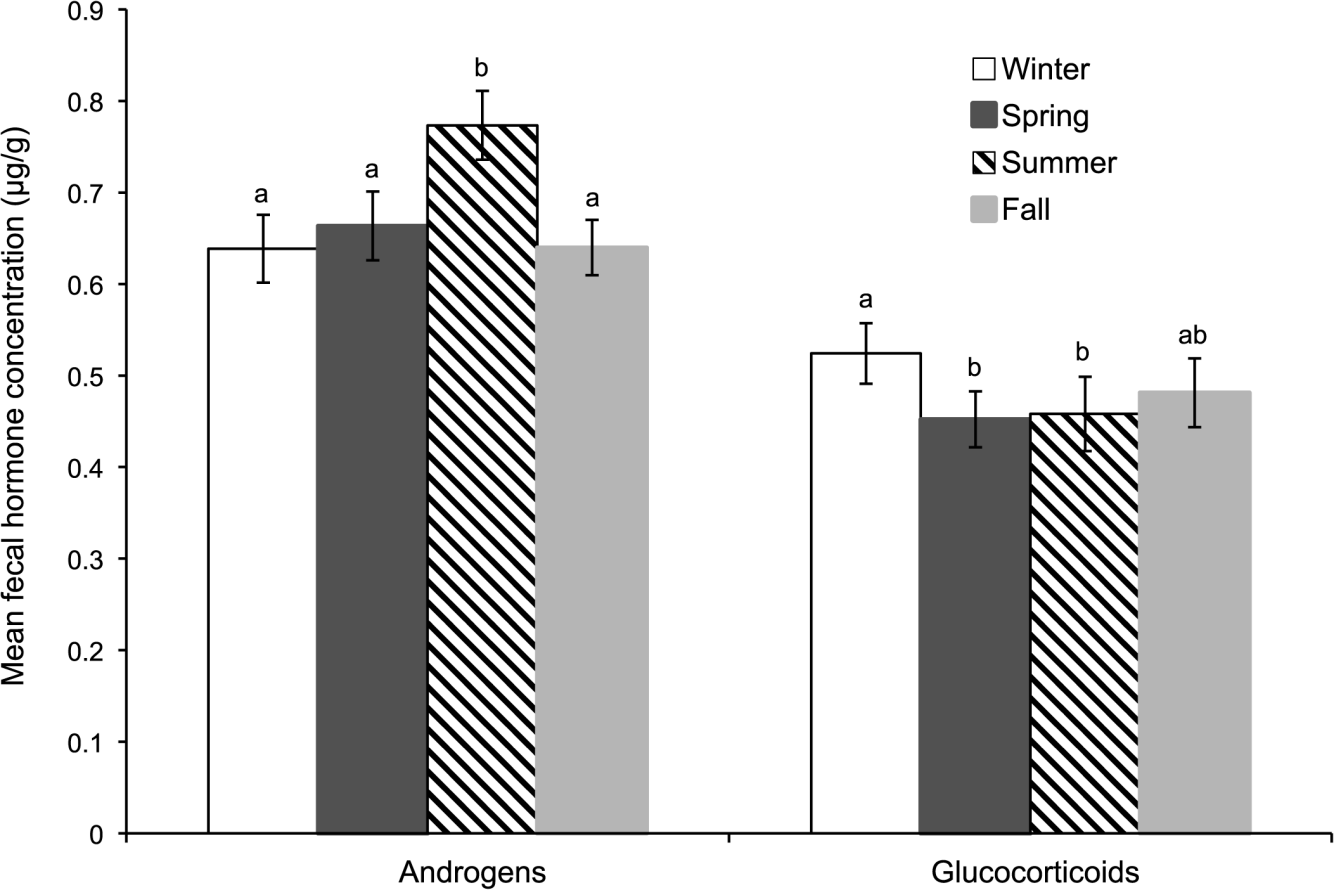
Mean (± SEM) fecal glucocorticoid and androgen metabolite concentrations in male cheetahs (n = 29) managed in North American zoological institutions across seasons. Within hormonal metabolite, different superscripts represent differences (*P* < 0.05) in hormonal concentrations.

For the 29 males in the present study, the mean androgen metabolite concentration was 0.69 ± 0.04 μg/g (range, 0.30–1.10 μg/g), and the baseline was 0.49 ± 0.02 μg/g (range, 0.21–0.82 μg/g). The mean glucocorticoid concentration for these same samples was 0.51 ± 0.04 μg/g (range, 0.21–1.08 μg/g) and the baseline 0.24 ± 0.02 μg/g (range, 0.12–0.52 μg/g). When evaluated seasonally, excreted androgen values were elevated (F_3,28_ = 22.2; *P* < 0.01) in the North American summer compared to any other season (Fig 3), whereas fecal glucocorticoid concentrations were higher (F_3,28_ = 15.6; *P* < 0.01) during the winter than in spring or summer.

**Fig 4:**
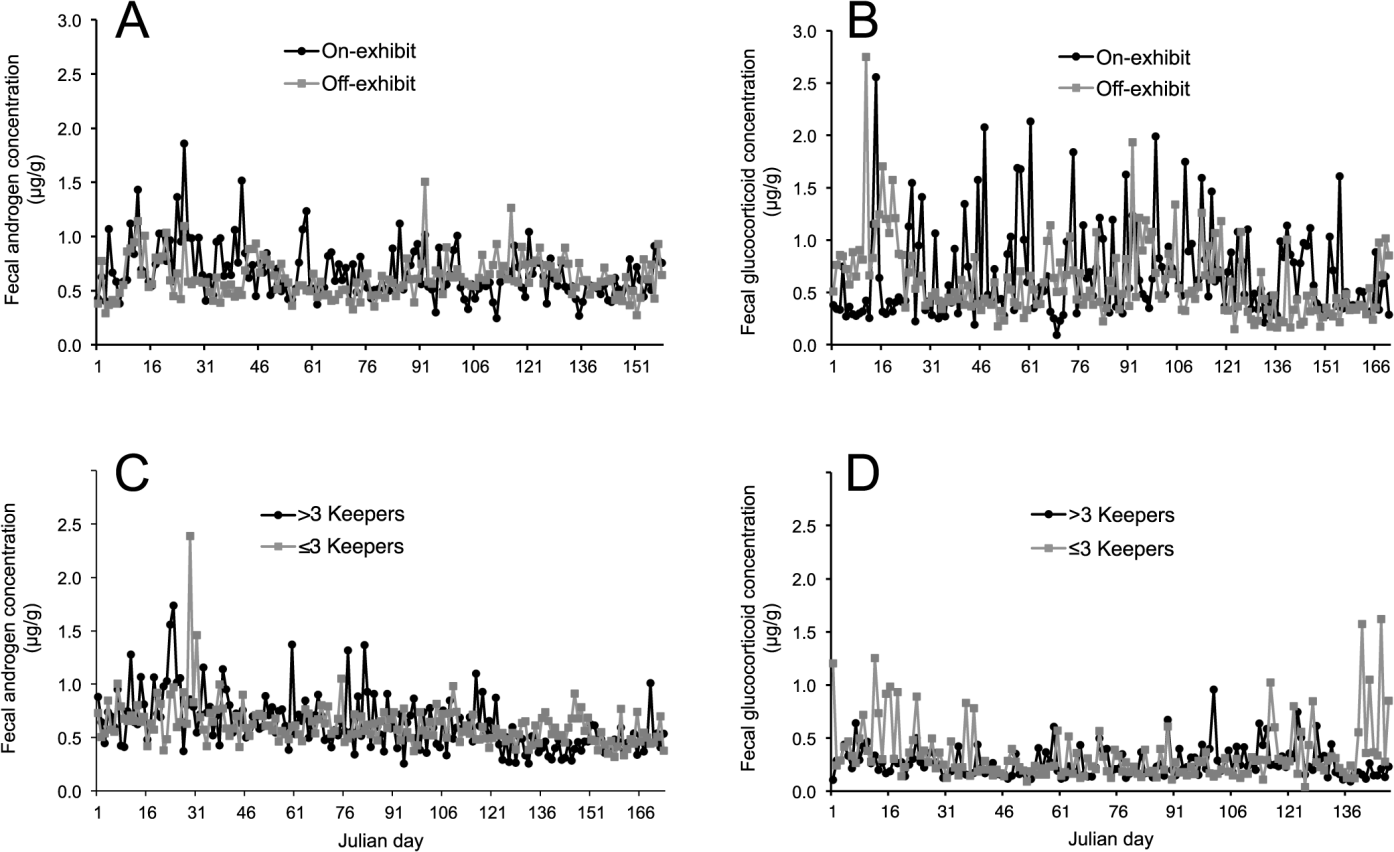
Fecal androgen (A, C) and glucocorticoid (B, D) metabolite profiles of two randomly selected, representative male cheetahs on the basis of on- or off public exhibit (A, B) or number of care-givers (C, D).

In the context of the treatment conditions, there was no direct influence (*P* > 0.05) of on- versus off-exhibit, number of care-givers, or number of conspecifics on excretion levels of either androgen or glucocorticoid metabolites (Table 3). This lack of effect evident from analyzing mean values also was reflected after plotting individual male patterns over time. For example, androgen and glucocorticoid metabolite profiles are depicted in representative males based on the on- or off-display (Fig 4A and 4B) or keeper number (Fig 4C and 4D) factor. No clear trends were apparent for either hormone, in part, because some males demonstrated marked day-to-day variation in metabolite excretion (Fig 4B).

**Table 3:** Gonadal and adrenal hormone metabolite concentrations for male cheetahs (n = 29) under different treatment conditions.

Treatment condition	Androgen metabolite concentration (μg/g)		Glucocorticoid metabolite concentration (μg/g)	
	Mean ± SEM	Baseline ± SEM	Mean ± SEM	Baseline ± SEM
On-exhibit (n = 12)	0.77 ± 0.05	0.52 ± 0.04	0.42 ± 0.05	0.20 ± 0.02
Off-exhibit (n = 17)	0.61 ± 0.04	0.47 ± 0.03	0.57 ± 0.05	0.26 ± 0.03
>3 Keepers (n = 13)	0.73 ± 0.04	0.52 ± 0.03	0.60 ± 0.06	0.31 ± 0.03
≤3 Keepers (n = 16)	0.63 ± 0.04	0.47 ± 0.03	0.43 ± 0.04	0.18 ± 0.01
>11 Conspecifics (n = 14)	0.69 ± 0.05	0.50 ± 0.03	0.53 ± 0.06	0.26 ± 0.03
≤11 Conspecifics (n = 15)	0.66 ± 0.04	0.48 ± 0.03	0.48 ± 0.05	0.21 ± 0.02

Number of cheetahs in each condition indicated in parentheses.

When Pearson correlation analyses were applied, there were no relationships (*P* > 0.05) between any ejaculate or endocrine metrics: mean or baseline androgen metabolite concentrations; mean, baseline, or peak frequency in glucocorticoid excretion; or the seminal traits of ejaculate volume, sperm concentration, total spermatozoa/ejaculate, motility factors, total motile spermatozoa/ejaculate, or percent structurally-normal spermatozoa/ejaculate. Male age, however, did positively correlate with mean (*r* = 0.48; *P* = 0.01) and baseline (*r* = 0.48; *P* = 0.01) glucocorticoid concentration. Age did not correlate (*P* > 0.05) with any other ejaculate or endocrine metric.

### Behavioral evaluations

All behavioral states were grouped into two principal components with eigenvalues of >1, together accounting for 64% of the total variation (S4 Table). The states standing, walking, pacing, running, and negative resting were grouped as the first principal component, and these were collectively termed ‘active states’. Sitting and crouching states were grouped as the second principal component and together were considered ‘inactive states’. Behavioral event frequencies analyzed by PCA yielded four principle components with eigenvalues of >1, together accounting for 66% of the total variation (S5 Table).

**Fig 5:**
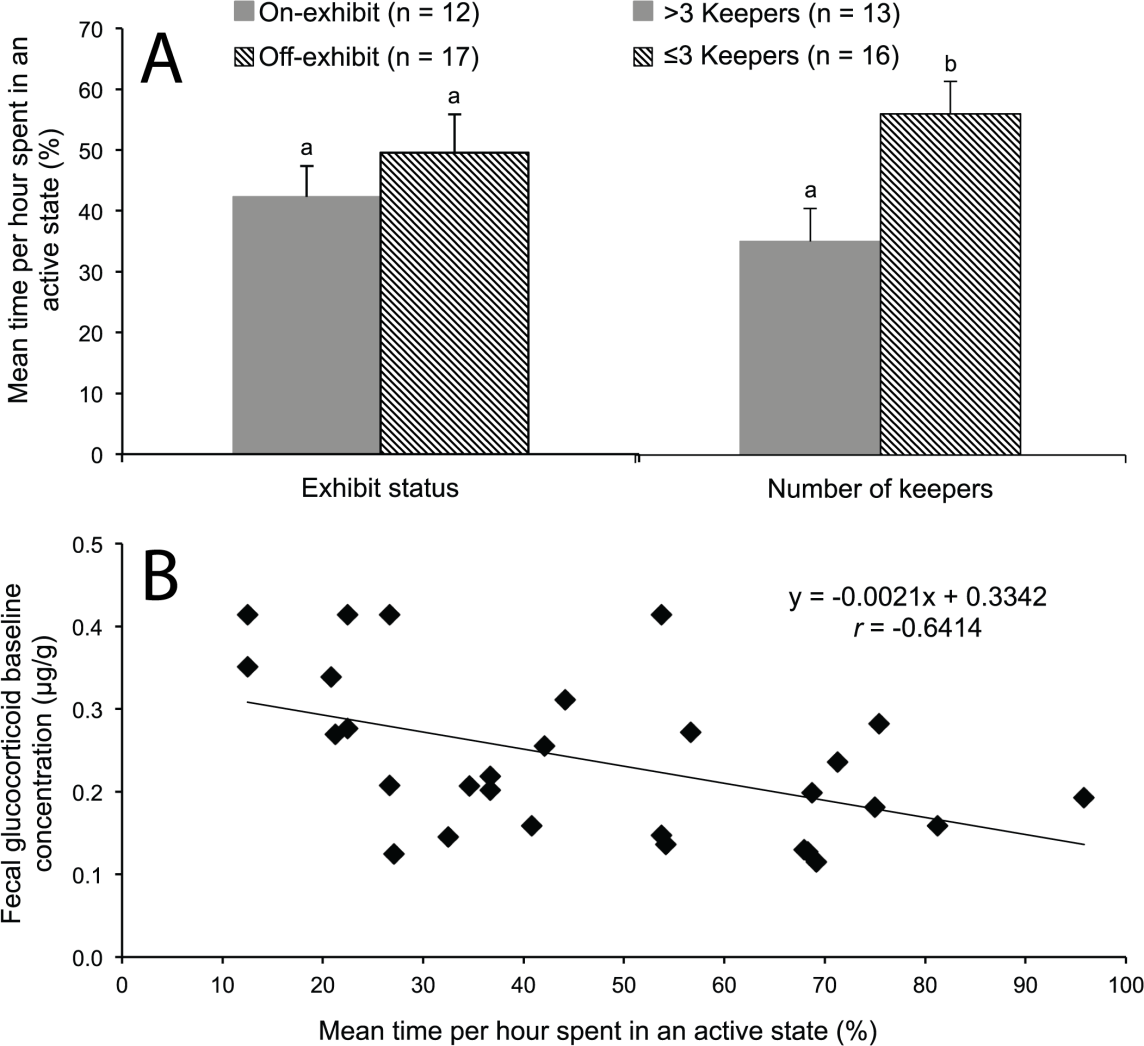
(A) Mean time per h male cheetahs (n = 29) spent in an active behavioral state based on being on- or off-exhibit or in the presence of >3 or ≤3 keeper care-givers. (B) Relationship between mean time males spent in an active state versus baseline fecal glucocorticoid concentration. For Panel **A**, different superscripts represent differences (*P* < 0.05).

Only our main factor of number of care-givers influenced cheetah behavior. Males housed in institutions with ≤3 keepers on staff spent a greater proportion of time (t_16_ = 3.91; *P* < 0.01) in an active state (component score, 0.41 ± 0.36; combined raw data in Fig 5A, 56.0 ± 5.4%) than counterparts in institutions with >3 keepers (component score, -0.33 ± 0.40; combined raw data in Fig 5A, 35.0 ± 5.4%). There was no difference (*P* > 0.05) in time spent in an active state between cheetahs housed on- or off-exhibit (Fig 5A) or between males housed at institutions with ≤11 conspecifics or >11 conspecifics in nearby enclosures. Among all males, the mean proportion of time spent in an active state was negatively correlated with baseline (*r* = -0.64; *P* < 0.01) and mean (*r* = -0.42; *P* = 0.02) glucocorticoid metabolite concentrations (Fig 5B). There were no differences (*P* > 0.05) in behavioral events between cheetahs living in different conditions (on- versus off-exhibit, number of keepers, or number of conspecifics). Post-hoc analysis within our statistical model of the proportion of time each male spent pacing (isolated from other PCA-grouped ‘active states’) yielded no difference (*P* > 0.05) in pacing behavior between facilities that varied in exhibit status, number of keepers, or number of conspecifics. There were no other correlations (*P* > 0.05) between any behaviors and seminal metrics, hormonal values or patterns, or age of individual males.

## Discussion

The cheetah consistently (1) produces many sperm pleiomorphisms in low sperm density ejaculates and (2) reproduces poorly in *ex situ* conditions. Although the species has little genetic variation (due to two historic population bottlenecks [5, 6, 39]), the lack of heterozygosity does not fully explain the unique seminal features or challenging reproductive performance [40]. What is clear is that cheetahs managed in North American zoos do not comprise a self-sustaining population. Unless the keys to consistent reproduction can be determined, computer modeling has predicted that the *ex situ* insurance population will continue to decline towards extinction [41]. Therefore, there is a need to prospectively study as well as mine existing databases to understand those factors that are influencing reproductive success in this rare species. Our approach was to exploit the highly predictable sperm phenotype that has been studied for >30 years [29] and the assertions by others that the cheetah may be stress-sensitive [15, 42]. Using this information, we examined the influence of three environmental conditions (on- versus off-exhibit, number of care-givers, and number of conspecifics in the same facility) on seminal characteristics and androgen/glucocorticoid patterns. We also took advantage of the rich history of ethological studies of this species in nature [14, 43–46] and zoos [17, 22, 38, 47–49], with those methods incorporated into our investigation. Using this multidisciplinary approach, we discovered that exposing cheetahs to traditional on-exhibit conditions as well as to >3 care-givers profoundly reduced sperm output (the total numbers of motile spermatozoa per ejaculate) and overall behavioral activity. None of our metrics were influenced by number of nearby cheetahs in the same facility.

The mechanism by which the exhibit experience and number of keepers affected sperm output and behavior was unrelated to either altered androgen or glucocorticoid metabolite levels or patterns. Based on an analysis of >4,600 fecal samples from 29 males, we found no direct connection of either of these hormones to any of the treatment conditions or seminal metrics. The one exception was an observed relationship between elevated glucocorticoids and specific stress-related behaviors, indicating a possible hormonal relationship to too many animal care-givers. It was expected that androgen metabolite profiles would be unrelated to sperm quality, as a lack of association has been reported for other felid species. For example, serum testosterone concentrations are similar between free-living and captive-managed Iberian lynx (*Lynx pardinus*), even though these two populations differ in testis weight, sperm motility traits, and acrosomal integrity [50]. Free-ranging African lions (*Panthera leo*) with less genetic diversity ejaculate more malformed spermatozoa than nearby, more heterozygous conspecifics, although both groups produce similar circulating testosterone [51]. Likewise, some domestic cats (*Felis catus*) consistently produce more sperm pleiomorphisms than others, but with no differences in peripheral androgen levels [52].

There also was no evidence that the compromised sperm quality in cheetahs maintained on-display in urban zoos was related to hyper-adrenal activity. Glucocorticoid concentrations and patterns were ubiquitous across treatment groups, which was unexpected given arguments by others that this species is sensitive to stress-inducing extrinsic factors [15, 16]. The latter position largely has been supported by findings of higher fecal glucocorticoid excretion and increased adrenal cortex size in captive-managed compared to free-ranging cheetahs in nature [15]. There also has been some effort to associate purported stress vulnerability in the cheetah to a host of chronic, degenerative pathologies found almost exclusively in captivity, including veno-occlusive disease [42, 53,54], glomerulosclerosis [42, 53,54], and amyloidosis [42,53]. However, despite analyzing thousands of samples with a rigorously validated assay, we failed to observe differences in glucocorticoid patterns between cheetahs managed on- or off-exhibit. Of course, it is well established that a naturally wild animal living in *ex situ* conditions and then exposed to a novel environmental factor will react with increased glucocorticoid production [55–57]. Although glucocorticoids often decline over the course of days or weeks as the individual ‘habituates’, this reduction may be due to internal mechanisms that keep stress hormones low even though physiological effects still can be expressed [58,59]. At least for felids, such a reaction can be species-specific. For example, individuals of the behaviorally-shy clouded leopard (*Neofelis nebulosa*) exposed to high people density and other adjacent species under *ex situ* conditions excrete chronically elevated glucocorticoid metabolites that are ~300% lower in counterparts housed off-display in a private area [60]. No such situational difference was observed for the cheetah, and the lack of any distinguishing differences may have been related to significant intra-individual variations measured in glucocorticoid (and androgen) profiles from day-to-day (Fig 4). This variability has been observed earlier in cheetahs and appears common to daily functionality rather than linked to any specific disruptor of adrenal activity [17].

Therefore, although observed sperm output differences were not influenced via androgen or glucocorticoid mediators, presence of cheetahs in an urban-based, exhibit setting while cared for by too many (>3) keepers was detrimental to sperm production and behavioral activities. Because we studied animals across seven institutions with diverse artificial habitats and enclosure sizes, it was impossible to examine the role of other environmental elements (e.g., total space available, physical distance to the public, presence/absence of other species and their density) on results. We only focused on a minimum number of unfamiliar people visiting the viewing/management area and the total number of care-givers. Obviously, exhibit visitors and their highly variable numbers, proximity, and generated noise and distractions introduce significant uncertainty for animals. Likewise, the more keepers providing care, the less opportunity a cheetah has to become familiar with a given care staff member. Unpredictability becomes a component of daily life, with an associated inability to understand what is ‘normal’ or to form keeper-animal bonds known to be important for successfully managing healthy, viable species within zoos [61]. Therefore, although there were multiple factors potentially influencing our sperm output and behavioral activity findings, the common element appeared to be capricious contact with unfamiliar people.

Although the effect on ejaculate characteristics was not mediated directly through androgen or glucocorticoid production, it is possible that the perturbations were expressed through other hormonal routes. For example, group housing is used routinely to induce a physiological state of chronic stress in male laboratory mice [62]. While having enlarged adrenals, the circulating glucocorticoid concentrations in these mice are no different from unstressed counterparts [63]. It has been postulated that many physiological effects of chronic stress occur not from altering the traditional hypothalamic-pituitary-adrenal axis, but rather via up-regulation and increased sensitivity of specific tissues to the posterior pituitary-released hormone, arginine vasopressin (AVP) [64, 65]. A connection between AVP and the testis, including decreased sperm number and motility, has been identified from studies of the rat [66, 67], mouse [68, 69], and human [70]. Such mechanisms likely exist in other mammals like the cheetah and deserve exploration as a potential means by which exposure to many unfamiliar people can be detrimental to ejaculate quality.

That cheetah sperm output was influenced by exposure to people was bolstered further when we examined historical data from an even larger male cohort (>100) known to be living either in an on-exhibit, urban zoo versus an off-display breeding center. Again, the latter individuals produced two- to three-fold more spermatozoa than the former. Besides being statistically significant, we suggest that this discovery is functionally relevant, especially as the cheetah produces relatively few total spermatozoa compared to other large felids evaluated similarly (e.g., African lion [71], tiger [*Panthera tigris*] [8], leopard [8]). Additionally, ~75% of cheetah spermatozoa are morphologically-deformed [7–11] (as also confirmed in the present study), and it is well established that these cells do not participate in fertilization [10, 12, 72]. Although the number of motile, normally-formed spermatozoa needed for conception to natural mating is unknown, the success of artificial insemination (AI) in the cheetah is reduced when the inseminate contains <16 x 10^6^ total motile spermatozoa [73]. As cheetah spermatozoa are sensitive to cryopreservation, >30 x 10^6^ total motile sperm/ml are required for AI when freeze-thawing is a component of the breeding strategy [7, 20, 74–77]. Further, the ability to produce embryos via *in vitro* fertilization is sperm-dosage dependent [78]. Collectively, this means that sperm number indeed matters in the cheetah. Most importantly, our findings demonstrated that some males were being managed under conditions that were marginalizing sperm production, perhaps to below standards essential for reproductive success.

Certain aspects of cheetah behavior were affected, but only by number of institutional care-givers. The use of fewer total keepers resulted in a higher frequency of standing, walking, pacing, and running behaviors. While there was no association of any of the people metrics (including keeper number) within our model on overall glucocorticoid excretion concentrations or patterns, it was possible to associate the latter hormone with the amount of behavioral activity using a correlation analysis. Specifically, cheetahs that were more behaviorally dynamic were excreting less glucocorticoid. This finding was interesting in the context of earlier observations by Carlstead and colleagues [79] who examined activity, behaviors, and excreted cortisol in the leopard cat (*Prionailurus bengalensis*), a small-size felid known to be highly sensitive to its captive surroundings [79]. Leopard cats exposed to novel environments and barren enclosures produce higher adrenal hormone production, attempt to hide more, and decrease exploratory behaviors [79]. Elevated glucocorticoid excretion also has been observed in clouded leopards that demonstrate more stationary behaviors, such as sleeping and hiding [60]. Collectively, these observations suggest that glucocorticoid excretion profiles, as an indicator of adrenal function, may be associated more closely to certain behaviors rather than reflecting an always-challenging and ambiguous classification of ‘stress’. Although our present study did not detect a hyper-adrenal response in cheetahs provoked by imposed treatments, some perhaps less marked glucocorticoid fluctuations appeared to be occurring that were linked to certain behaviors. This was logical as glucocorticoids play important roles in behavioral manifestations in animals, sometimes occurring acutely after exogenous administration [80]. Generally, this adrenal-behavioral relationship is oriented to survival tactics and addressing adverse environmental conditions. For example, glucocorticoids are believed to be a driver in territory abandonment behaviors in some bird species, while generally maintained in low concentrations during the breeding season to prevent desertion of the nest or offspring [80, 81]. Thus, it was possible that amounts of glucocorticoids were influencing cheetah behaviors, as observed in the present study, a hypothesis that could be tested by more intensive monitoring of both hormonal patterns and selective behaviors under varying environmental conditions.

The one major element studied that did not influence seminal, endocrine, or behavioral results in our studied males was the presence of other cheetahs, either males or females. This was interesting given that female ovarian cyclicity is markedly influenced by the immediate presence of even one other cheetah of the same gender. Wielebnowski and colleagues [17] found that pairs of some cheetahs mutually suppressed ovarian activity while increasing expression of distress-linked behaviors and inter-animal aggression. When animals in the pair were split up into adjacent enclosures separated by a simple fenceline that retained visual/olfactory proximity, each female resumed ovarian activity. The authors concluded that ‘grouping’ (as small as pairing) could shut down gonadal function, a finding perhaps explaining significance of the solitary nature of the species in the wild [14]. By contrast, it is well known that free-ranging male cheetahs can comprise close-knit, kinship groups of two to four members [14, 18]. Therefore, it would be logical for males to have evolved a different mechanism from females, in that presence of conspecifics has no impact on gonadal activity, including sperm output. In this way, male cheetahs in groups or adjacency always would retain maximal capacity for fertility, thus increasing chances for reproduction. Likewise, it makes sense that we observed no evidence of male seasonality in cheetahs managed under controlled, *ex situ* conditions. Male reproductive aseasonality also has been reported for cheetahs living in captivity in an African range country [11], and cheetah studbook data reveals births at breeding institutions occurring in every month of the year. Although we observed circannual consistency in seminal quality in our study animals, there were elevations in androgen metabolites in summer and glucocorticoids in winter. The ~20% increase in androgens, while significant, was modest compared to three- to four-fold increases that occur in true seasonally-breeding felid species (e.g., snow leopard [*Panthera uncia*] [28] and Pallas’ cat [*Otocolobus manul*] [27]), where gonadal activity and sexual behavior are restricted exclusively to a certain time of the year. The glucocorticoid increases in winter also were not surprising, having been observed in other wildlife taxa (e.g., carnivores [82] and artiodactyl [83, 84]) and associated with a seasonal need for (among others) energy mobilization to ensure survival during an energetically-costly time of year [80].

Our conclusion that sperm output and behavioral activity are compromised in cheetahs living in an *ex situ* environment exposed to significant numbers of people (both visitors and care-givers) has practical implications. The finding that >90% of cheetah offspring have been produced in off-display facilities already has begun to influence the structure of the breeding program for this species in North America. For example, since 2012, cheetah adults identified as the most genetically valuable have been moved from traditional urban zoos into spacious centers, most of which have no (or minimal) visitor exposure. Based on our findings here, this strategy also should help maintain higher sperm outputs especially if these off-exhibit facilities additionally rely on a smaller, dedicated keeper staff to promote familiarity with daily routines. Such expansive environments that can maintain far more cheetahs than in a typical city zoo also allow opportunities to conduct more hypothesis-based research, such as contemporary priorities associated with mate choice, onset of puberty, and how to diagnose pregnancy noninvasively during the first trimester of gestation. Meanwhile, present findings demonstrate that, at least for the cheetah, environmental/management conditions influence physiological function as well as behaviors. Application of findings to improve best-practice management protocols will advance the field of conservation breeding for achieving a self-sustaining *ex situ* cheetah population.

## Supporting Information

S1 Fig Fecal androgen metabolite concentrations in a male cheetah given a GnRH agonist (deslorelin) implant (s.c.). Solid horizontal lines indicate mean androgen concentration before and after implant insertion.
(TIF)

S2 Fig Serum cortisol concentrations in adult cheetahs (n = 7) after administration (time 0) of exogenous adrenocorticotrophic hormone (ACTH; one of two dosages and a total of 5 treatment events) or saline (3 control events). Different superscripts represent differences (*P* < 0.05) in cortisol concentrations among injection times.
(TIF)

S3 Fig Mean (± SEM) ejaculate traits for male cheetahs (n = 29) managed in *ex situ* collections that were housed at institutions with >11 or ≤ 11 conspecifics present (number/group indicated in parentheses). There were no differences (*P* > 0.05) in any trait between groups.
(TIF)

S1 Table Fecal glucocorticoid metabolite concentrations measured daily in adult cheetahs before and after administration of exogenous ACTH. (DOCX)

S2 Table Ejaculate traits for male cheetahs managed in *ex situ* collections that were housed on- or off-exhibit. (DOCX)

S3 Table Percentages of specific structural deformities of spermatozoa from cheetahs managed in *ex situ* collections that were housed on- versus off-exhibit. (DOCX)

S4 Table Component loadings for principal components analysis of behavioral state variables for 29 adult male cheetahs. (DOCX)

S5 Table Component loadings for principal components analysis of behavioral event variables for 29 adult male cheetahs. (DOCX)

S1 Text Testosterone EIA validation description and results. (DOCX)

S2 Text Cortisol EIA validation description and results. (DOCX)
