# Supplementary figures and images for: Motile Sperm Output by Male Cheetahs (Acinonyx jubatus) Managed Ex Situ Is Influenced by Public Exposure and Number of Care-Givers
Source: PLoS One. 2015 Sep 2;10(9):e0135847. doi: 10.1371/journal.pone.0135847 (PMC4558051; doi:10.1371/journal.pone.0135847)

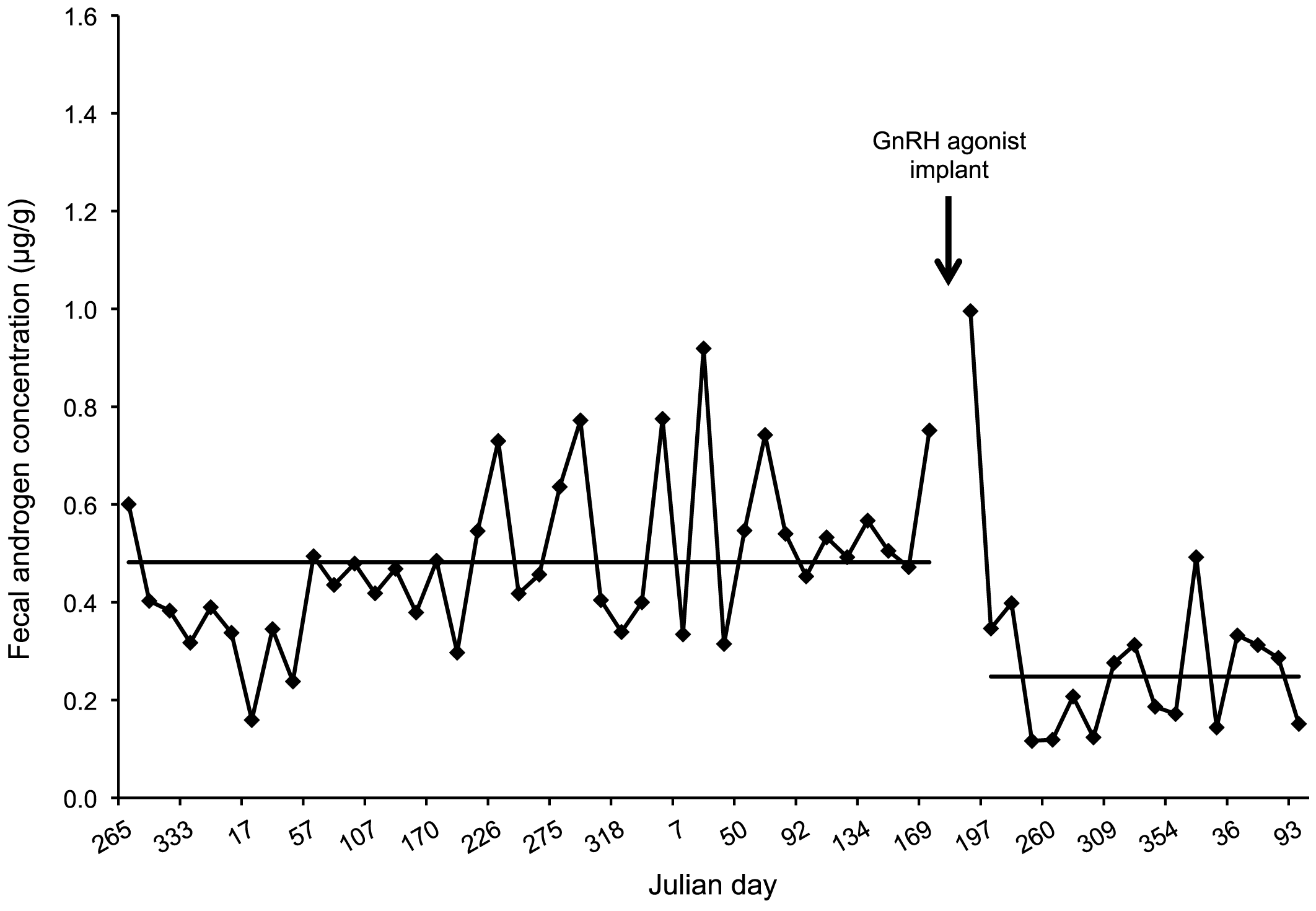

Supplement: S1 Fig — Solid horizontal lines indicate mean androgen concentration before and after implant insertion. (TIF) [file pone.0135847.s001.tif]

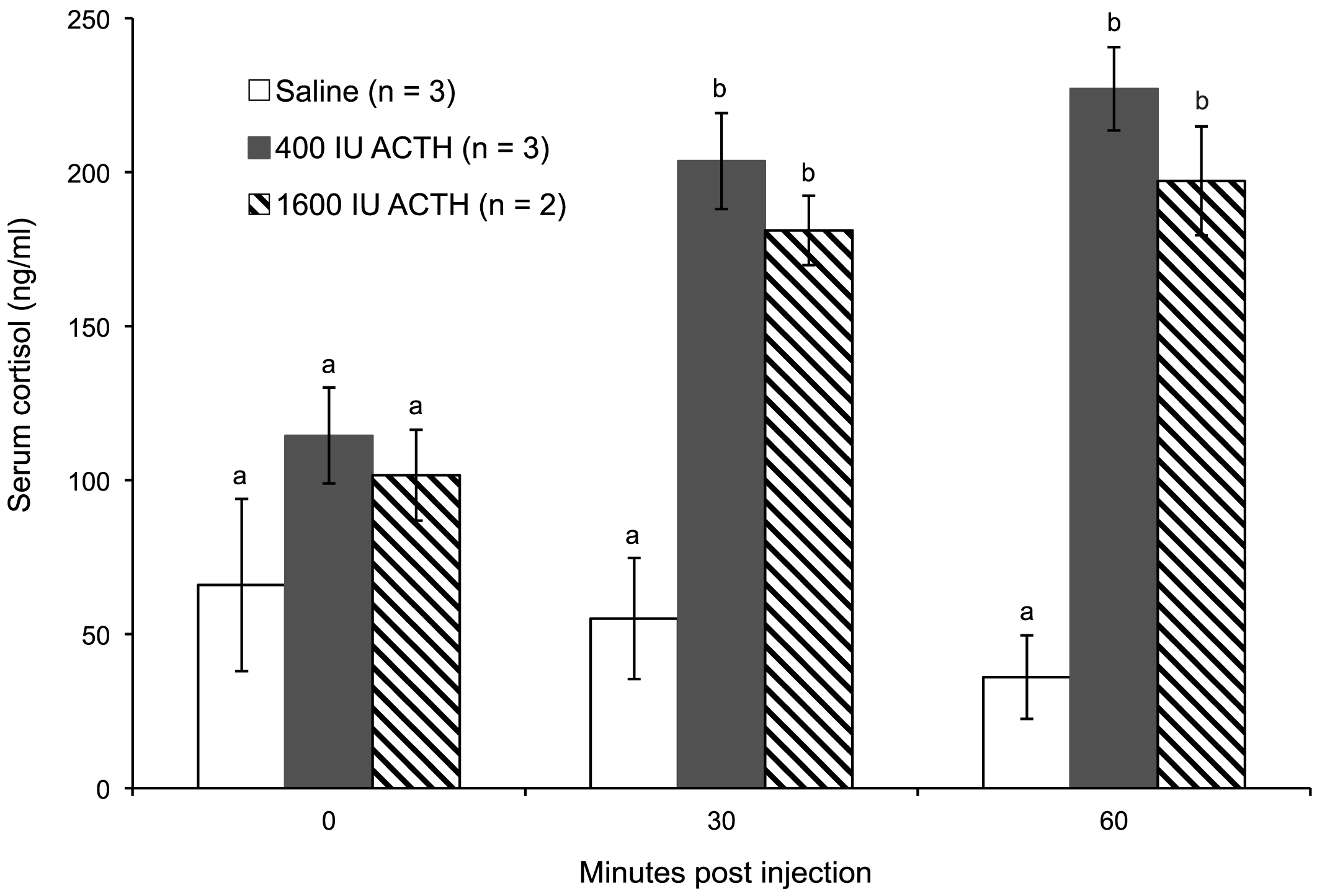

Supplement: S2 Fig — Different superscripts represent differences (P < 0.05) in cortisol concentrations among injection times. (TIF) [file pone.0135847.s002.tif]

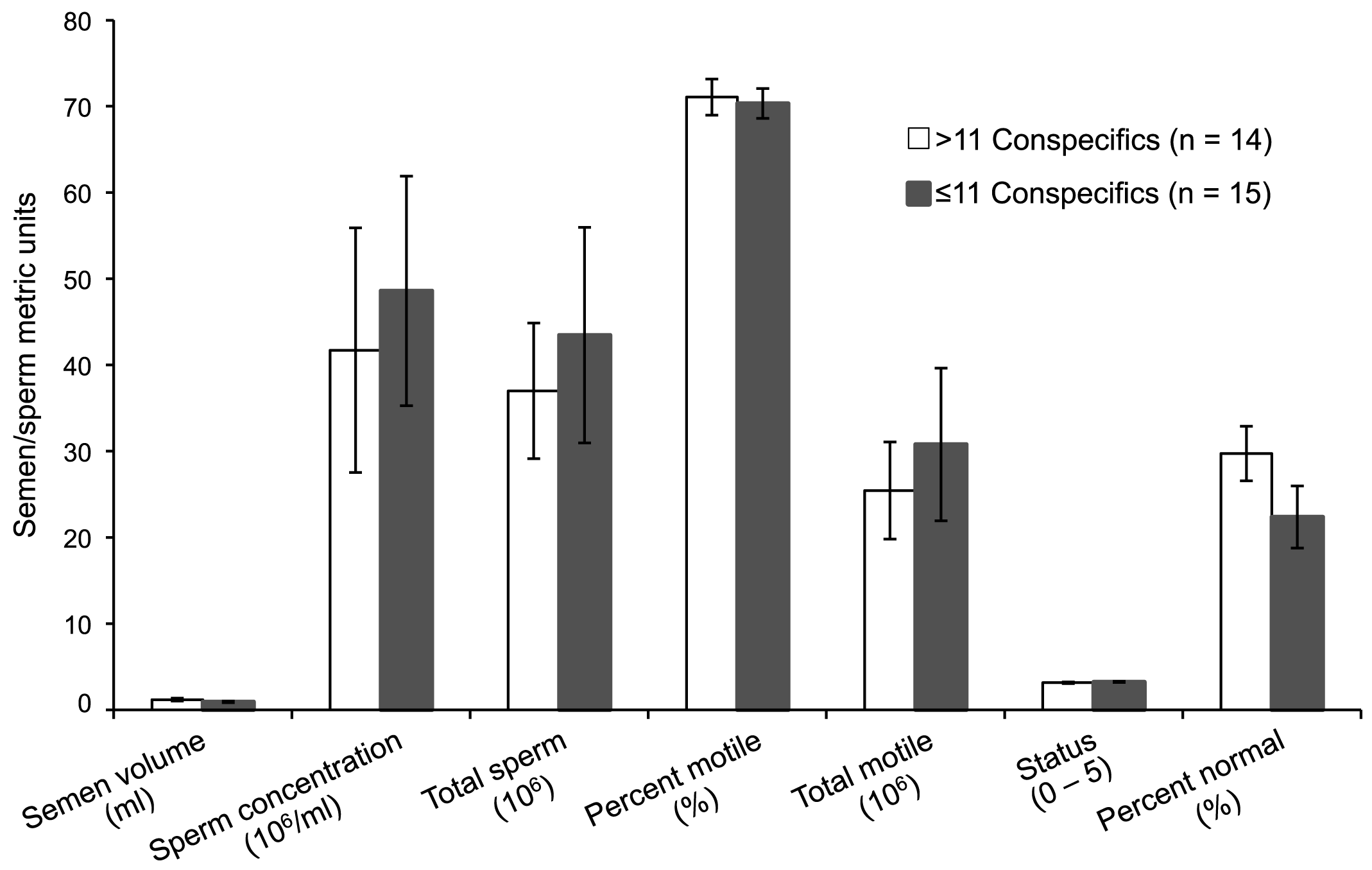

Supplement: S3 Fig — There were no differences (P > 0.05) in any trait between groups. (TIF) [file pone.0135847.s003.tif]
