# Supplementary material for: Motile Sperm Output by Male Cheetahs (Acinonyx jubatus) Managed Ex Situ Is Influenced by Public Exposure and Number of Care-Givers
Source: PLoS One. 2015 Sep 2;10(9):e0135847. doi: 10.1371/journal.pone.0135847 (PMC4558051; doi:10.1371/journal.pone.0135847)
Supplement: S1 Table — (DOCX) [file pone.0135847.s004.docx]

| Animal no. | ACTH dose (IU) | Glucocorticoid metabolite concentration (μg/g) | | Rise^d^ (%) | Hours between treatment and peak |
| --- | --- | --- | --- | --- | --- |
|  |  | Pre-treatment mean^a^ (± SEM) | Peak^b,c^ |  |  |
| M1 | 400 | 0.28 ± 0.05 | 0.58 | 208 | 48 |
| M2 | 400 | 0.28 ± 0.05 | 0.33 | 117 | 48 |
| F1 | 400 | 0.19 ± 0.02 | 0.98 | 521 | 96 |
| F2 | 1600 | 0.35 ± 0.01 | 1.11 | 318 | 24 |
| M3 | 1600 | 0.18 ± 0.02 | 0.29 | 161 | 72 |

Immunoreactive metabolites in feces were measured with a cortisol-3-carboxymethyloxime EIA.

^a^ Represents the mean glucocorticoid metabolite concentration for all pre-treatment samples including the day of injection.

^b^ Refers to the single sample containing the ACTH-induced peak in glucocorticoid metabolites.

^c^ Increased (*P* = 0.02) over pre-treatment mean concentrations.

^d^ Represents the peak expressed as a percentage of the pre-treatment mean.
