# Supplementary material for: Motile Sperm Output by Male Cheetahs (Acinonyx jubatus) Managed Ex Situ Is Influenced by Public Exposure and Number of Care-Givers
Source: PLoS One. 2015 Sep 2;10(9):e0135847. doi: 10.1371/journal.pone.0135847 (PMC4558051; doi:10.1371/journal.pone.0135847)
Supplement: S2 Table — (DOCX) [file pone.0135847.s005.docx]

| Trait | Male collection | On-exhibit | Off-exhibit |
| --- | --- | --- | --- |
| Testicular volume (cm^3^) | Current study | 11.2 ± 0.4 | 11.0 ± 0.5 |
|  | Historical database | 13.6 ± 0.6 | 13.8 ± 0.6 |
| Semen volume (ml) | Current study | 0.96 ± 0.14 | 1.11 ± 0.13 |
|  | Historical database | 1.56 ± 0.10 | 1.46 ± 0.14 |
| Percent motile (%) | Current study | 71.7 ± 2.4 | 70.0 ± 1.6 |
|  | Historical database | 66.5 ± 1.5 | 69.65 ± 1.7 |
| Forward progressive status (0 – 5) | Current study | 3.2 ± 0.1 | 3.2 ± 0.1 |
|  | Historical database | 3.2 ± 0.1 | 3.3 ± 0.1 |
| Percent normal (%) | Current study | 28.8 ± 4.3 | 23.9 ± 2.9 |
|  | Historical database | 20.4 ± 1.7 | 24.4 ± 2.9 |

Data are reported from the current study (n = 23 males; n = 8 on-exhibit, n = 15 off-exhibit) or from those in our institution’s historical database (n = 159 ejaculates; n = 116 on-exhibit, n = 43 off-exhibit). Traits expressed as mean ± SEM. There were no differences (*P* > 0.05) in traits between on- or off-exhibit males.
