# Supplementary material for: Motile Sperm Output by Male Cheetahs (Acinonyx jubatus) Managed Ex Situ Is Influenced by Public Exposure and Number of Care-Givers
Source: PLoS One. 2015 Sep 2;10(9):e0135847. doi: 10.1371/journal.pone.0135847 (PMC4558051; doi:10.1371/journal.pone.0135847)
Supplement: S3 Table — (DOCX) [file pone.0135847.s006.docx]

| Structural deformity | On-exhibit (n = 8) | Off-exhibit (n = 15) |
| --- | --- | --- |
| Macrocephalic | 0.4 ± 0.2 | 0.4 ± 0.2 |
| Microcephalic | 10.1 ± 3.2 | 7.52 ± 1.5 |
| Bicephalic | 0.1 ± 0.1 | 0.2 ± 0.1 |
| Abnormal acrosome | 20.8 ± 4.0 | 19.3 ± 2.4 |
| Abnormal midpiece | 1.6 ± 0.5 | 1.9 ± 0.5 |
| No midpiece | 0.2 ± 0.2 | 0.2 ± 0.1 |
| Tightly coiled tail | 1.4 ± 0.4 | 3.5 ± 1.4 |
| Biflagellate | 0.2 ± 0.1 | 0.1 ± 0.1 |
| Bent midpiece with droplet | 19.3 ± 2.2 | 20.2 ± 2.3 |
| Bent midpiece without droplet | 1.3 ± 0.6 | 1.1 ± 0.4 |
| Bent tail with droplet | 2.4 ± 0.8 | 3.6 ± 1.0 |
| Bent tail without droplet | 0.7 ± 0.3 | 2.4 ± 1.4 |
| Proximal droplet | 5.7 ± 1.9 | 7.1 ± 2.7 |
| Distal droplet | 1.6 ± 0.4 | 1.1 ± 0.5 |
| Spermatid | 5.3 ± 2.1 | 7.6 ± 2.4 |

Specific structural deformities are expressed as mean percentage of the total deformed cells ± SEM. There were no differences (*P* > 0.05) in the proportion any deformity between on- or off-exhibit males.
