# Supplementary material for: Motile Sperm Output by Male Cheetahs (Acinonyx jubatus) Managed Ex Situ Is Influenced by Public Exposure and Number of Care-Givers
Source: PLoS One. 2015 Sep 2;10(9):e0135847. doi: 10.1371/journal.pone.0135847 (PMC4558051; doi:10.1371/journal.pone.0135847)
Supplement: S4 Table — (DOCX) [file pone.0135847.s007.docx]

|  | Component 1 | Component 2 |
| --- | --- | --- |
| Variance explained | 0.39 | 0.25 |
| Label | Active states | Inactive states |
| Resting | **-48** | -40 |
| Standing | **40** | -33 |
| Crouching | -2 | **68** |
| Sitting | 18 | **36** |
| Walking | **45** | -36 |
| Pacing | **47** | 6 |
| Running | **39** | 6 |

Values are multiplied by 100 and rounded to the nearest integer. Bolded numbers indicate the highest loading component score for each variable.
