# Supplementary material for: Motile Sperm Output by Male Cheetahs (Acinonyx jubatus) Managed Ex Situ Is Influenced by Public Exposure and Number of Care-Givers
Source: PLoS One. 2015 Sep 2;10(9):e0135847. doi: 10.1371/journal.pone.0135847 (PMC4558051; doi:10.1371/journal.pone.0135847)
Supplement: S5 Table — (DOCX) [file pone.0135847.s008.docx]

|  | Component 1 | Component 2 | Component 3 | Component 4 |
| --- | --- | --- | --- | --- |
| Variance explained  Label | 0.21  Body marking | 0.19  Interest | 0.14  Urine marking | 0.12  Wash/threaten |
| Rub | **43** | 27 | -23 | 12 |
| Roll | **47** | -3 | 18 | 22 |
| Sniff | 29 | **44** | 6 | -18 |
| Groom | 38 | 1 | -38 | **43** |
| Stutter | -15 | **47** | 29 | 28 |
| Meowchirp | **-41** | 10 | 20 | 17 |
| Growl | -15 | 34 | 22 | **53** |
| Tread | 26 | -32 | **45** | -4 |
| Urinate | 18 | -39 | **47** | 29 |
| Urine Spray | 18 | 29 | **34** | -34 |
| Defecate | 16 | 22 | 21 | **-37** |

Values are multiplied by 100 and rounded to the nearest integer. Bolded numbers indicate the highest loading component score for each variable.
