# Supplementary material for: Motile Sperm Output by Male Cheetahs (Acinonyx jubatus) Managed Ex Situ Is Influenced by Public Exposure and Number of Care-Givers
Source: PLoS One. 2015 Sep 2;10(9):e0135847. doi: 10.1371/journal.pone.0135847 (PMC4558051; doi:10.1371/journal.pone.0135847)
Supplement: S1 Text — (DOCX) [file pone.0135847.s009.docx]

## Testosterone EIA validation

Biological validation of the testosterone EIA was determined in a preliminary study by comparing androgen metabolite concentrations in 29 adult male cheetahs (>2 yr in age) to seven counterpart males (<2 yr) and six adult females (>2 yr). A secondary validation involved another male cheetah (1 yr in age at initiation of sample collection) where fecal samples were collected biweekly for a 21 mo period before and then for 9 mo after the s.c. insertion of a deslorelin (Suprelorin; 18.8 mg) implant. Deslorelin is a GnRH agonist well established to suppress testicular function, including in the cheetah [[1](#_ENREF_1), [2](#_ENREF_2)].

## High performance liquid chromatography analysis

Here, we examined number and relative proportions of androgen metabolites in cheetah fecal extracts using reverse-phase high-performance liquid chromatography (HPLC) with a protocol developed for the domestic cat [[3](#_ENREF_3)]. In brief, prior to HPLC, samples were reconstituted in 0.5 ml phosphate buffer (pH, 7.0), passed through a C-18 matrix column (Spice Cartridge, Rainin, Woburn, MA), and eluted with 5 ml of 80% methanol to remove contaminants (sample loss ~10%). The filtered fecal extract was evaporated to dryness, reconstituted in 60 μl acetonitrile, and eluted from a Microsorb C-18 column (Rainin) using a gradient of 45% acetonitrile isocratic over 80 to 120 min (1 ml/min flow rate, 333 μl fractions). Fractions were dried under pressurized air, reconstituted in BSA-free phosphate buffer (pH, 7.0), and androgen metabolite immunoreactivity was then quantified by EIA (as described above). Co-elution profiles of ^3^H-testosterone, ^3^H-androstenedione, and ^3^H-dihydrotestosterone reference tracers (39,416 dpm) also were determined.

Ether extractions and enzyme hydrolysis were performed to determine the proportion of ether-soluble, water-soluble, and hydrolyzable androgen metabolites [[4](#_ENREF_4)]. Extracted samples in BSA-free phosphate buffer were extracted again with 10 volumes of diethyl ether to separate water-soluble from ether-soluble forms. Residual aqueous samples were enzymatically-hydrolyzed with 50 μl β-glucuronidase/aryl sulfatase (20,000 Fishman U/40,000 Roy U, respectively; Boehringer Mannheim Corp., Indianapolis, IN) at 37°C for 24 h, and then extracted again with 10 volumes of diethyl ether to separate enzyme-hydrolyzable (organic phase) from non-hydrolyzable (aqueous phase) forms.

## Statistical analysis

To ensure biological validity of androgen assessments, we first compared androgen metabolite concentrations in adult males versus young males and adult females using a one-way analysis of variance (ANOVA) procedure followed by a Tukey-Kramer multiple mean comparison test [[5](#_ENREF_5)]. The second validation was associated with the GnRH agonist delivery experiment and involved calculating mean androgen metabolite concentration and involved assessment before and after deslorelin implantation in a single male, with values compared using a paired *t*-test.

## Results

The assay for androgen metabolites was determined valid on the basis of the following findings. First, mean values were higher (*P* < 0.01) in adult (>2 yr) males (0.69 ± 0.04 μg/g dry feces) compared to young (<2 yr) males (0.39 ± 0.02 μg/g) or adult (>2 yr) females (0.29 ± 0.03 μg/g). Secondly, after producing a pre-treatment baseline of 0.48 ± 0.03 μg/g, the male implanted with deslorelin produced a brief androgen metabolite spike within 4 d of s.c. placement (to 1.0 μg/g; S1 Fig.) followed by a mean concentration (0.25 ± 0.03 μg/g) that was lower (*P* < 0.01) than pre-implantation. HPLC analysis revealed three to four androgen metabolites in feces, little of which corresponded with the ^3^H-labelled androgen reference tracers. Immunoreactivity was primarily associated with presumably conjugated androgens eluting earlier at fractions 8 to 16, similar to that reported earlier for the domestic cat and Eurasian and Iberian lynx [[3](#_ENREF_3), [6](#_ENREF_6)]. Androgen metabolites in cheetah feces were primarily water-soluble (57.5%), with 34.2% of the immunoreactivity found in the ether fraction after extraction. Of the water-soluble (presumably conjugated) forms, <5% were enzyme-hydrolyzable, similar to findings in the domestic cat [[3](#_ENREF_3)].

## References

1. Bertschinger HJ, Trigg TE, Jochle W, Human A. Induction of contraception in some African wild carnivores by down-regulation of LH and FSH secretion using the GnRH analogue deslorelin. Reproduction. 2002: 41-52.

2. Bertschinger HJ, Jago M, Nothling JO, Human A. Repeated use of the GnRH analogue deslorelin to down-regulate reproduction in male cheetahs (*Acinonyx jubatus*). Theriogenology. 2006;66: 1762-1767.

3. Brown JL, Terio KA, Graham LH. Fecal androgen metabolite analysis for non-invasive monitoring of testicular steroidogenic activity in felids. Zoo Biol. 1996;15: 425-434.

4. Brown JL, Wasser SK, Wildt DE, Graham LH. Comparative aspects of steroid hormone metabolism and ovarian activity in felids, measured non-invasively in feces. Biol Reprod. 1994;51: 776-786.

5. Grafen A, Hails R. Modern Statistics for the Life Sciences. 1st edn. New York, NY: Oxford University Press Inc.; 2002.

6. Jewgenow K, Naidenko SV, Goeritz F, Vargas A, Dehnhard A. Monitoring testicular activity of male Eurasian (*Lynx lynx*) and Iberian (*Lynx pardinus*) lynx by fecal testosterone metabolite measurement. Gen Comp Endocrinol. 2006;149: 151-158.
