# Supplementary material for: Motile Sperm Output by Male Cheetahs (Acinonyx jubatus) Managed Ex Situ Is Influenced by Public Exposure and Number of Care-Givers
Source: PLoS One. 2015 Sep 2;10(9):e0135847. doi: 10.1371/journal.pone.0135847 (PMC4558051; doi:10.1371/journal.pone.0135847)
Supplement: S2 Text — (DOCX) [file pone.0135847.s010.docx]

## Cortisol EIA validation

Number and relative proportions of glucocorticoid metabolites in cheetah fecal extracts have been determined earlier using reverse-phase high-performance liquid chromatography (HPLC) [[1](#_ENREF_1)]. To confirm the biological relevance of glucocorticoid metabolite data, a preliminary adrenocorticotropic hormone (ACTH) challenge study was conducted in seven adult cheetahs at SCBI. Three were designated as saline controls (2 males, 1 female). The remaining four plus one male previously used (3 mo earlier) as a saline control were treated with ACTH (1 challenge each). While anesthetized (as described above) for a routine veterinary examination, each animal was administered one i.m. injection of saline or ACTH (Corticotrophin LA; 80 units/ml; 400 or 1,600 IU; Wedgewood Pharmacy, Swedesboro, NJ) which was designated Day 0. To monitor effect, both blood and fecal samples were taken before and after treatment administration. For the former, a 7 ml aliquot of blood was drawn by needle aspiration from a leg vein within 5 min of treatment injection (Time 0) and then again at 30 and 60 min post-administration. Serum was harvested within 1 h after blood sample centrifugation (1,000*g*, 20 min) and then frozen-stored until analyzed (as described above). Fecal sampling commenced 5 to 10 d before ACTH injection (Day 0) and continued daily until Days 5 to 11 with samples packaged and stored as described above. Serum cortisol concentrations for all samples were grouped and averaged by collection time point (Time 0, 30 min, 60 min) and by dosage (saline versus 400 IU or 1,600 IU ACTH). For fecal results, individual pre-treatment averages were calculated for each cheetah as the mean of fecal samples collected on all days before the ACTH challenge, including Day 0. Adrenocortical responses to ACTH for each individual were expressed as a percentage of the pre-treatment average, with the average value being equivalent to 100%. Post-response averages also were calculated for each cheetah as the mean concentration for all samples collected after the observed adrenocortical response peak.

## Statistical analysis

Glucocorticoid assay validity was determined on the basis of the ACTH challenge results. For assessments in serum, we examined cortisol concentration differences among collection time points using a repeated measures ANOVA followed by a Tukey-Kramer multiple mean comparison test with a simple covariance matrix; a one-way ANOVA with Tukey-Kramer multiple mean comparison test accounted for ACTH dosage and the saline control. For fecal evaluations, a repeated measures ANOVA followed by a Tukey-Kramer multiple mean comparison test with a first-order autoregressive covariance matrix was used to determine differences between pre-treatment means, peak concentrations, and post-response means.

## Results

For the validation of adrenal hormone assays, serum cortisol concentrations increased (*P* < 0.01) at 30 min after ACTH injection, but were not different (*P* > 0.05) between ACTH doses or between the 30 and 60 min post-injection time points (S2 Fig.). For evaluation in feces, glucocorticoid metabolite concentrations increased (*P* = 0.02) from 117 to 521% in all test animals to peak 1 to 4 d after ACTH administration and then declined to concentrations comparable (*P* > 0.05) to the pre-treatment mean within 24 h (S1 Table).

## References

1. Young KM, Walker SL, Lanthier C, Waddell WT, Monfort SL, Brown JL. Noninvasive monitoring of adrenocortical activity in carnivores by fecal glucocorticold analyses. Gen Comp Endocrinol. 2004;137: 148-165.
